# Increased ambulatory arterial stiffness index and blood pressure load in normotensive obese patients

**DOI:** 10.4314/ahs.v21i3.27

**Published:** 2021-09

**Authors:** Fatma Kaplan Efe, Mujgan Tek

**Affiliations:** 1 Internal Medicine; University of Health Sciences Kecioren Research and Training Hospital; 2 Tobb university medical school cardiology clinic, Cardiology; Tobb Etu Hastanesi

**Keywords:** Ambulatory arterial stiffness index, blood pressure load, obesity, blood pressure

## Abstract

**Objectives:**

It has been shown that blood pressure (BP) values measured in obese subjects are higher than the individuals with normal weight, even in normotensive limits. However, data concerning the Ambulatory Arterial Stiffness Index (AASI) and blood pressure load in normotensive obese subjects is lacking. This study was aimed to compare the ambulatory arterial stiffness index and blood pressure load in normotensive obese and healthy controls.

**Methods:**

One hundred normotensive obese and one hundred normal weight subjects were included in this study. All subjects underwent 24-hour ambulatory blood pressure monitoring. Ambulatory arterial stiffness index was calculated from 24-hour ambulatory blood pressure monitoring records. Ambulatory arterial stiffness index was defined as one minus the regression slope of unedited 24-h diastolic on systolic blood pressures. Systolic blood pressure (SBP) and diastolic blood pressure (DBP) load values were calculated from 24-hour ambulatory blood pressure monitoring analysis.

**Results:**

Ambulatory arterial stiffness index of the obese subjects was significantly higher than the healthy controls (0.48±0.2 vs. 0.33±0.11, p<0.001). 24-hours systolic blood pressure and diastolic blood pressure loads were significantly higher in obese subjects. Logistic regression analysis revealed that body mass index (BMI) was an independent predictor for an abnormal ambulatory arterial stiffness ındex (≥0.50) (OR: 1.137, 95% CI: 0.915-1.001, p=0.004).

**Conclusion:**

Blood pressure load and ambulatory arterial stiffness index are increased in normotensive obese patients. Moreover, body mass index is an independent predictor for an abnormal ambulatory arterial stiffness index. Our results indicate that obese subjects are at higher risk for future cardiovascular events despite normal office BP levels.

## Introduction

Obesity, which may give rise to the development of various diseases, is currently one of the most important health problems globally. The prevention and early diagnosis of the elevated blood pressure (BP) may help reducing the complications associated with hypertension. The relationship between obesity and hypertension has been well established. The blood pressure values measured in obese patients are higher than normal weight subjects even in the normotensive range^1^. However, office BP measurements may be insufficient in detection of the elevated BP. Ambulatory blood pressure monitoring (ABPM) is therefore increasingly employed in diagnosis of the hypertension in subjects with obesity^2^. However, studies conducted until now have frequently used ABPM in defining type and stage of hypertension, or to evaluate circadian rhythm in obese patients. The Ambulatory Arterial Stiffness Index (AASI) and blood pressure load are additional indices derived from ABPM records^3^–^5^. AASI has been shown to be a strong predictor of cardiovascular disease and target organ damage in hypertensive patients^6^, ^7^. Blood pressure load has also been found in association with the severity of the hypertension and target organ damage^8^, ^9^.

The aim of our study was to evaluate the AASI and BP load in obese patients who were diagnosed as having normal BP on office BP measurements.

## Methods

Obese patients (BMI≥30) aged >18 years, without overt hypertension, and whose office blood pressure lower than 140/80 mmHg were underwent ABPM at TOBB Economics and Technology University Hospital and Kecioren Teaching and Research Hospital, Ankara. ABPM results were evaluated according to European Society of Cardiology (ESC) hypertension guidelines^10^, and obese patients with ambulatory normotension were included the study. The control group consisted of 100 healthy, non-obese individuals with BMI lower than 25, who underwent 24-hours ambulatory BP monitoring for suspected HT.

All subjects underwent transthoracic echocardiography as a part of the routine cardiovascular examination. The office blood pressure of each patient in the study was measured by manual sphygmomanometers on the left arm after 5 minutes of rest. Three consecutive blood pressure measurement averages were obtained for participants with systolic below 140 mmHg and diastolic below 90 mmHg. ABPM monitoring was performed with a commercially available ABPM device (Suntech AccuWin ProV3, Suntech medical, Inc. Morrisville, NC, USA). Automatic BP recordings were obtained regularly every 30 minutes during the 24-hour ABPM period. Patients with mean 24-hour systolic BP (SBP) ≥ 130 mmHg and/or diastolic BP (DBP) ≥80 mmHg, mean daytime SBP≥135 mmHg and/or DBP≥85 mmHg and mean nighttime SBP>120 mmHg, and nighttime DBP≥70 mmHg on ABPM were diagnosed as hypertensive, and below this values were defined as normotensive^10^, ^11^. Nighttime and daytime periods were selfreported. The change in nocturnal BP decline was calculated automatically. A regression slope of diastolic over systolic BP was computed for each participant. AASI was defined as 1 minus the regression slope^12^. An AASI ≥ 0.50 was accepted abnormal^4^. Blood pressure load was defined as the percentage of BP values reaching or exceeding 135 mmHg SBP or 85 mmHg DBP during daytime and 120 mmHg SBP or 70 mmHg DBP during nighttime on 24-hour blood pressure readings. According to systolic blood pressure (SBP) dropping pattern during night-time compared with daytime, patients were classified into extreme dipper (≥20%), dipper (≥10%, <20%), nondipper (≥0%, <10%), and reverse dipper (BP rises during night-time).

Body mass indexes (BMI) were calculated using the formula BMI: kg/m^2^. Obesity is classified as a BMI ≥30 kg/m^2^ 13.

Demographic, clinical and laboratory parameters including glucose, creatinine, lipid profile and complete blood count parameters were recorded in all patients. Informed consent was obtained from each patient and the study was approved by the institutional ethics committee.

The differences in AASI and BP load between the obese subjects and healthy controls were the primary outcome measure of the study. The predictive role of BMI on AASI was the secondary outcome measure.

### Statistical analysis

All statistical analyses were performed using the SPSS 25 (SPSS INC, Chicago, Illinois, USA). Categorical variables were expressed as frequencies and continuous variables as mean ± standard deviation. The independent samples t test was used to compare continuous variables and chi-square test was used to compare categorical variables between the groups. Correlation analysis was performed to assess the association between the interventricular septum thickness (IVS) and AASI and BP load. Logistic regression analysis was performed to identify the contributors of an abnormal AASI (≥ 50). Variables demonstrating a statistical significance of <0.01 on univarate analysis were included in the logistic regression model. A p value <0.05 was considered statistically significant.

## Results

**Figure 1:**
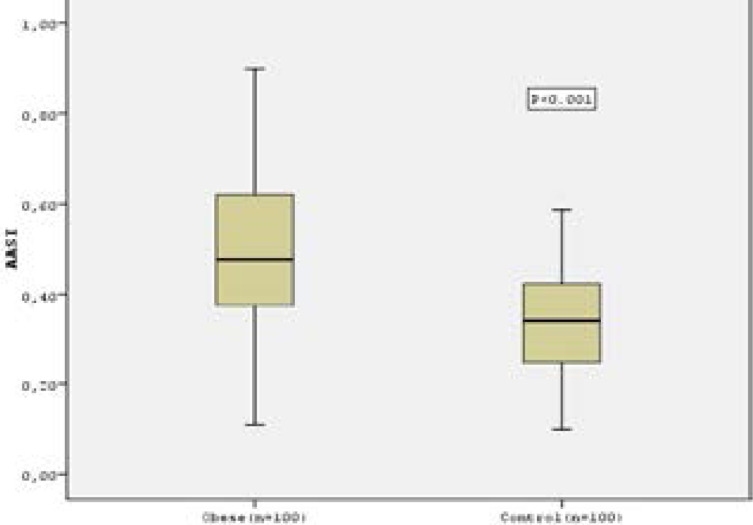
Ambulatory Arterial Stiffness Index in obese subjects and healthy controls

Baseline clinical and laboratory data of the groups are shown in Table 1. Blood glucose levels, interventricular septal thickness and left ventricular end-diastolic diameter were significantly higher in obese subjects (101.5±22.7 m/dl vs. 90.4±13.04 mg/dl, 0.95±0.09 cm vs. 0.82±0.11 cm, 4.6±0.26 cm v. 4.4±0.37 cm, respectively, p<0.05 for all). Comparison of the ABPM parameters of the groups is shown in Table 2. SBP and DBP have been found increased, both in daytime and night time, in obese subjects compared to healthy controls. 24-hours SBP and DBP loads were significantly higher in obese subjects compared to healthy controls. AASI of the obese subjects was also significantly higher than that of the healthy controls (0.48±0.2 vs. 0.33±0.11, p<0.001, Figure 1). The number of non-dipping subjects was similar in the two groups (70 % vs. 56%, p=0.125). However, neither AASI nor the BP load were correlated with left ventricular end-diastolic diameter.

**Table 1:** Comparison of demographic information, anthropometric measures and laboratory parameters of groups

	Obese (n=100)	Control (n=100)	P
Age (years)	44.8±9.5	41.2±10.6	0.055
Gender(F/M)	78/32	77/23	0.309
BMI (kg/m2)	34.9±5.4	22.3±2.1	>0.001
Hemoglobin	13.8±1.5	13.7±1.4	0.805
Glucose (mg/dL)	101.5±22.7	90.4±13.04	0.005
T.cholesterol (mg/dL)	207.8±33.5	198.5±44.4	0.311
LDL cholesterol (mg/dL)	128.3±30.09	118.09±29.7	0.143
HDL cholesterol (mg/dL)	49.3±14.8	53.2±17.9	0.316
Triglyceride (mg/dL)	156.6±76.7	148.7±79.7	0.681
Creatinine (mg/dL)	0.8±0.15	0.75±0.13	0.086
TSH	2.07±1.2	2.1±3.4	0.904
IVS thickness, cm	0.95±0.09	0.82±0.11	0.001
LVEDD, cm	4.6±0.26	4.4±0.37	0.034
EF, %	63.7±0.26	64.8±7.1	0.571

BMI: Body Mass Index, IVS: interventricular septum LDL cholesterol: low-density lipoprotein cholesterol, HDL cholesterol: high-density lipoprotein cholesterol,

**Table 2:** Comparison of ABPM parameters of the study groups

	Obese(n=100)	Control(n=100)	p
**24- h SBP, mmHg**	122.32±6.8	114.2±7.9	0.001
**Daytime SBP,** **mmHg**	124.53±7.4	116.69±8.2	<0.001
**Nighttime SBP,** **mmHg**	115.42±7.3	106.1±9.3	<0.001
24 -h DBP, mmHg	73.9±6.2	69.6±7.1	0.001
**Daytime DBP,** **mmHg**	75.7±6.6	71.7±7.7	0.004
**Nightime DBP,** **mmHg**	67.8±5.6	62.15±7.7	<0.001
**24-h SBP load, (%)**	17.7±11.1	6.5±8.2	<0.001
**Daytime SBP load,** **(%)**	12.7±11.3	4.5±7.04	<0.001
**Nighttime SBP** **load,(%)**	32.5±24.4	12.8±16.1	<0.001
**24-h DBP load, (%)**	9.8±11.7	5.8±9.5	0.046
**Nighttime DBP** **load,(%)**	9.2±14.1	5.7±11.1	0.151
**AASI**	0.48±0.2	0.33±0.11	<0.001

**AASI**; ambulatory arterial stiffness index

**Figure 2:**
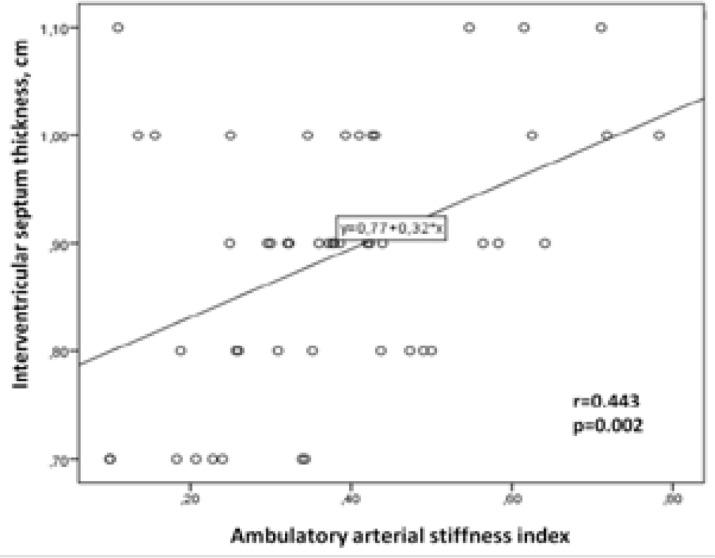
The correlation between interventricular septum thickness and ambulatory arterial stiffness index

Logistic regression analysis revealed that BMI was an independent predictor for an abnormal AASI (≥0.50) (OR: 1.137, 95% CI: 0.915-1.001, p=0.004). AASI was significantly correlated with the IVS (r=0.443, p=0.002, Figure 2). However, no significant correlation was observed between IVS and systolic and diastolic BP loads (r=0.250, p00.083, and r=0.23, p=0.877, respectively).

## Discussion

In our study we showed for the first time that BP load and AASI are increased in normotensive obese patients compared to healthy controls. Moreover, we found that BMI is an independent predictor for an abnormal AASI. Given the critical predictive role of AASI on future cardiovascular events, our findings suggests that the risk for developing hypertension and cardiovascular events may be increased in obese population even if their BP is in normal range. AASI is an easily available marker of arterial stiffness, which can be calculated by the commercially available ABP monitoring devices. AASI has been proven to be associated with cardiovascular adverse events and vascular damage in several clinical settings^14^. The Dublin Outcome Study has shown that AASI is likely to be a more potent predictor of stroke than pulse pressure, particularly in normotensive individuals^15^. Saner et al have reported that AASI is elevated in obese children and mainly influenced by BMI independent from the systolic and diastolic blood pressure values^3^. The mechanism of increaed arterial stiffness in obese children has been explained by the activation of sympathetic nervous system and endothelial dysfunction^16^, ^17^. Similar to the findings reported by Saner et al., we found that BMI is as independent predictor for an abnormal AASI (≥0.50) in adult population.

Blood pressure load which has been defined as the proportion of BP readings above set thresholds, is frequently employed in indicating the severity of the BP elevation. It has been shown that subjects with increased BP load are at high risk for developing hypertension despite a normal BP level^18^, ^19^. Previous studies demonstrated the association between BP load and target organ damages, including left ventricular hypertrophy, increased arterial stiffness and nephropathy^9^, ^20^. BP load has also been suggested as an index of BP variability for normotensive individuals^3^, ^9^. BP load has also been shown to predict future cardiovascular events^21^. In the present study we observed higher systolic and diastolic BP load in obese subjects than the healthy controls. Our results indicate that despite normal office BP subjects with obesity have higher AASI and BP load compared to healthy controls. With this in mind, we may suggest that obese subjects with normal office BP measurements are at high risk for future cardiovascular events, hypertension and end-organ damage.

Our study has some limitations. First we analyzed the data that were collected retrospectively from our medical records. Waist circumference of the patients was not available, and we could not analyze the association with central obesity. We have no data about the duration of obesity. Absence of some metabolic profiles (eg. insulin, HOMA index) or lack of data about sleep quality or sleep disorders could be other limitations. There is strong and well known association between sleep disorders, hypertension and obesity. Both obesity and sleep disorders like obstructive sleep apnea (OSA) are risk factors for the development hypetension.^22^

## Conclusion

Blood pressure load and AASI are increased in normotensive obese patients compared to healthy controls. Moreover, BMI is an independent predictor for an abnormal AASI. Given the critical predictive role of AASI on future cardiovascular events, our findings may suggest that the risk for developing hypertension and cardiovascular events is increased in obese subjects with normal office blood pressure measurements.
